# Supplementary material for: Internet-based peer support interventions for people living with HIV: A scoping review
Source: PLoS One. 2022 Aug 30;17(8):e0269332. doi: 10.1371/journal.pone.0269332 (PMC9426879; doi:10.1371/journal.pone.0269332)
Supplement: S1 Appendix — Protocol developed by the researchers following the Open Science Framework guidelines. (DOCX) [file pone.0269332.s001.docx]

**S1 Appendix. Pre registration Protocol at Open Science Framework.**

**Internet-based peer support interventions for people living with HIV: A Scoping Review**

**PROTOCOL**

Prepared for Registration to Open Science.

Framework Submitted 10/03/2022.

**1.Review Title and Timescale**

**1.1. Review title:** Internet-based peer support interventions for people living with HIV: A

Scoping Review

**1.2. Anticipated or actual start date:** 10/03/2022.

**1.3. Anticipated completion date:** 31/03/2022.

**1.4. Stage of review at time of this submission:** Preliminary searches; Piloting of the study

selection process and Formal screening of search results against eligibility criteria.

**2.Review team details**

**2.1. Named contact:** Dr. Stefanella Costa-Cordella

**2.2. Named contact email:** stefanella.costa@udp.cl

**2.3. Named contact address:** Vergara 275, Santiago, Chile.

**2.4. Organizational affiliation of the review:** 1) Facultad de Psicología, Universidad Diego Portales. Santiago, Chile and 2) Fundación Arriarán. Santiago, Chile.

**2.5. Review team members and their organizational affiliations:**

| **Title** | **First name** | **Last name** | **Affiliations** |
| --- | --- | --- | --- |
| PhD | Stefanella | Costa-Cordella | Centro de Estudios en Psicología Clínica y Psicoterapia (CEPPS), Facultad de Psicología, Universidad Diego Portales. Santiago, Chile.  Instituto Milenio Depresión y Personalidad (MIDAP). Santiago, Chile.  Centro de Estudios en Neurociencia Humana y Neuropsicología (CENHN), Facultad de Psicología, Universidad Diego Portales. Santiago, Chile. |
| Ps | Aitana | Grasso-Cladera | Centro de Estudios en Psicología Clínica y Psicoterapia (CEPPS), Facultad de Psicología, Universidad Diego Portales. Santiago, Chile.  Centro de Estudios en Neurociencia Humana y Neuropsicología (CENHN), Facultad de Psicología, Universidad Diego Portales. Santiago, Chile. |
| PhD | Alejandra | Rossi | Centro de Estudios en Neurociencia Humana y Neuropsicología (CENHN), Facultad de Psicología, Universidad Diego Portales. Santiago, Chile. |
| PhD | Javiera | Duarte | Centro de Estudios en Psicología Clínica y Psicoterapia (CEPPS), Facultad de Psicología, Universidad Diego Portales. Santiago, Chile.  Instituto Milenio Depresión y Personalidad (MIDAP). Santiago, Chile. |
| MD | Flavia | Guiñazu | Web Intelligence Centre, Facultad de Ingeniería Industrial, Universidad de Chile, Santiago, Chile. |
| MD | Claudia P. | Cortes | Hospital Clínico San Borja Arriarán & Fundación Arriarán, Santiago, Chile.  Departamento de Medicina, Facultad de Medicina, Universidad de Chile, Santiago, Chile. |

**2.6. Funding sources/sponsors:** Founded detailed in the pre-registration form.

**2.7. Conflict of interest:** The authors declare that the research was conducted in the absence of any commercial or financial relationships that could be construed as a potential conflict of interest.

**3. Review methods**

**3.1. Review aims and question(s):** This scoping review aims to map the existing literature on psychosocial interventions for PLWHA based on peer support and delivered through the internet. The specific research questions are: 1) What internet-based peer support interventions are available for PLWHA? What are their main characteristics?, and 2) How do the available interventions understand peer support?

**3.2. Literature search:** Comprehensive literature searches of electronic bibliographic databases were conducted in PUBMED, Web of Science, and CINAHL complete (through EBSCO). The selection was made according to the institutional availability/access, for this reason some databases, such as EMBASE and Cochrane, were excluded. The search strategy was developed using the PRESS (Peer Review of Electronic Search Strategies) checklist. This step was conducted by the investigators due to institutional limitations. We will also scan the reference lists of all relevant reviews.

**3.3. Condition or domain being studied:** Psychosocial interventions for PLWHA based on peer support and delivered through the internet.

**3.4. Participants/Population:** People living with HIV and AIDS.

**3.5. Intervention(s)/Exposure(s):** Any psychosocial intervention (an activity used to modify

behavior and/or emotional state) designed specifically for PLWHA based on peer support and delivered through the internet. Peer support is defined as the support provided by people who share life experiences. Applied to interventions, peer support typically includes group meetings, support networks (either virtual or in-person) or peer-mentoring. Another concept commonly used to refer to peer support in the context of psychosocial interventions is peer group.

Internet delivery implies that the intervention is received through a device (such as computer or smartphone), using any service on the communications infrastructure of the Internet, such as the Web (including social network platforms such as facebook, blogs, forums, etc), email, instant messaging, voice calls, video calls (ref). Sometimes health services delivered through the internet are called: ehealth, mhealth, digital health, telemedicine, virtual health, mobile health and internet-based.

**3.6. Comparator(s) Control(s):** Any comparator is relevant for inclusion, such as studies comparing one form of peer support intervention with another peer support intervention or comparing peer support interventions with no peer support intervention, or comparing a face-to-face peer support intervention with an internet-based/digital/mobile health intervention. In addition, studies without a comparator are eligible for inclusion.

**3.7. Types of study to be included initially:** AII types of publications including published articles, articles in conference proceedings, editorials, websites, and chapters in textbooks are relevant.

**3.8. Context:** All periods of time and duration of follow-up are eligible.

**3.9. Primary outcome(s):** All primary outcomes are eligible.

**3.10. Secondary outcome(s):** All secondary outcomes are eligible.

**3.11. Data extraction (selection and coding):** Using the a priori eligibility criteria, a standardized questionnaire for study selection was developed for screening of titles and abstracts and full-text articles. Subsequently, three reviewers will screen citations and full-text articles for inclusion, independently. Inter-rater discrepancies will be resolved by discussion or a fourth person. We will extract data on characteristics of the articles (e.g., type of article or study), population characteristics (e.g., time since diagnosis, age, gender), intervention characteristics (e.g., how do they understand peer support, how do they deliver the intervention, which type of technology they use), and outcomes (e.g., adherence to treatment, quality of life, prevention of comorbidities).

**3.12. Risk of bias (quality) assessment:** Since this is a scoping review, we will not conduct quality appraisal, which is consistent with the framework proposed by Arksey and O'Malley, as well as the Joanna Briggs institute methodological guidance for Scoping Reviews.

**3.13. Strategy for data synthesis:** The synthesis will focus on providing: 1) A description of the existent psychosocial interventions based on peer-support and delivered through internet for PLWHA, and 2) A summary of the literature according to the types of interventions, participants (e.g., ages) ,comparators, and outcomes identified. Also, a summary of the different forms of peer support across the studies will be developed. Descriptive methods (i.e., frequencies, percentages) will be used to summarize.

**3.14. Analysis of subgroups or subsets:** Not applicable.

**4. Review general information**

**4.1. Type of review:** Scoping Review.

**4.2. Language:** English.

**4.3. Country:** Chile.

**4.4. Other registration details:**  Not applicable.

**4.5. Dissemination plans:** The manuscript reporting the present scoping review will be sent to be published in a relevant peer-review journal.

**4.6. Key words:** psychosocial interventions, peer support, digital health, HIV.

**4.7. Current review status:** In process: Preliminary searches; Piloting of the study selection

process and Formal screening of search results against eligibility cri
