# Supplementary material for: Internet-based peer support interventions for people living with HIV: A scoping review
Source: PLoS One. 2022 Aug 30;17(8):e0269332. doi: 10.1371/journal.pone.0269332 (PMC9426879; doi:10.1371/journal.pone.0269332)
Supplement: S2 Appendix — List of the eligibility criteria used to assess the articles for inclusion. (DOCX) [file pone.0269332.s002.docx]

**S2 Appendix. Eligibility Criteria.**

1. **Condition or domain being studied:** Psychosocial interventions for PLWHA based on peer support and delivered through the internet.
2. **Participants/Population:** People living with HIV and AIDS.
3. **Intervention(s)/Exposure(s):** Any psychosocial intervention (an activity used to modify behavior and/or emotional state) designed specifically for PLWHA based on peer support and delivered through the internet. Peer support is defined as the support provided by people who share life experiences. Applied to interventions, peer support typically includes group meetings, support networks (either virtual or in-person) or peer- mentoring. Another concept commonly used to refer to peer support in the context of psychosocial interventions is peer group.

Internet delivery implies that the intervention is received through a device (such as a computer or smartphone), using any service on the communications infrastructure of the Internet, such as the Web (including social network platforms such as Facebook, blogs, forums, etc), email, instant messaging, voice calls, video calls (ref). Sometimes health services delivered through the internet are called: ehealth, mhealth, digital health, telemedicine, virtual health, mobile health and internet-based.

1. **Comparator(s) Control(s):** Any comparator is relevant for inclusion, such as studies comparing one form of peer support intervention with another peer support intervention or comparing peer support interventions with no peer support intervention, or comparing a face-to-face peer support intervention with an internet-based/digital/mobile health intervention. In addition, studies without a comparator are eligible for inclusion.
2. **Types of study to be included initially:** AII types of publications including published articles, articles in conference proceedings, editorials, websites, and chapters in textbooks are relevant.
3. **Context:** All periods of time and duration of follow-up are eligible.
4. **Primary outcome(s):** All primary outcomes are eligible.
5. **Secondary outcome(s):** All secondary outcomes are eligible.
