# Supplementary material for: Internet-based peer support interventions for people living with HIV: A scoping review
Source: PLoS One. 2022 Aug 30;17(8):e0269332. doi: 10.1371/journal.pone.0269332 (PMC9426879; doi:10.1371/journal.pone.0269332)
Supplement: S3 Appendix — Full string of search implemented in PUBMED. The string of search was adapted to each database. (DOCX) [file pone.0269332.s003.docx]

**S3 Appendix. String of Search.**

("telemedicine"[MeSH Terms] OR (("digital"[All Fields] OR "digitalisation"[All Fields] OR "digitalised"[All Fields] OR "digitalization"[All Fields] OR "digitalize"[All Fields] OR "digitalized"[All Fields] OR "digitalizer"[All Fields] OR "digitalizing"[All Fields] OR "digitally"[All Fields] OR "digitals"[All Fields] OR "digitization"[All Fields] OR "digitizations"[All Fields] OR "digitize"[All Fields] OR "digitized"[All Fields] OR "digitizer"[All Fields] OR "digitizers"[All Fields] OR "digitizes"[All Fields] OR "digitizing"[All Fields]) AND "health*"[MeSH Terms]) OR "mobile health*"[MeSH Terms] OR (("virtual"[All Fields] OR "virtuality"[All Fields] OR "virtualization"[All Fields] OR "virtualized"[All Fields] OR "virtualizing"[All Fields] OR "virtuals"[All Fields]) AND "health*"[MeSH Terms]) OR "telemedicine"[MeSH Terms]) AND ("hiv"[MeSH Terms] OR "acquired immunodeficiency syndrome"[MeSH Terms]) AND (("peer"[All Fields] AND "support*"[MeSH Terms]) OR "peer group*"[MeSH Terms]).
